# Role of Aromatic Herbs and Spices in Salty Perception of Patients with Hyposmia

**DOI:** 10.3390/nu14234976

**Published:** 2022-11-23

**Authors:** Antonella Rosa, Francesco Loy, Ilenia Pinna, Carla Masala

**Affiliations:** Department of Biomedical Sciences, University of Cagliari, Cittadella Universitaria, SS 554, Km 4.5, 09042 Monserrato, Italy

**Keywords:** olfactory dysfunction, hyposmia, taste perception, chemoreception, salty perception

## Abstract

Herbs and spices represent a possibility for the improvement of anosmia and ageusia. In this work we evaluated the role of Mediterranean aromatic herbs and spices in the salty taste perception of patients with hyposmia compared to healthy controls. To this goal, the salty taste perception in response to pure salt and different types of commercial flavored sea salt was assessed in patients with hyposmia, with or without a post-acute coronavirus syndrome, and healthy controls. Myrtle berries and leaves, a mixture of Mediterranean herbs and plants such as helichrysum, rosemary, liquorice, fennel seeds and myrtle leaves, oranges and saffron were used as salt flavoring ingredients. Differences in gustatory perception between 57 patients with hyposmia and 91 controls were evaluated considering the rate of the gustatory dimensions of pleasantness, intensity, and familiarity, using a 7-point hedonic Likert-type scale. At a dose of 0.04 g/mL, saline solutions of flavored salts, with an average 15% less NaCl, were perceived by patients with hyposmia as equally intense but less familiar than pure salt solution, with similar scores in the pleasantness dimension. Our study highlighted the central role of Mediterranean aromatic plants in the enhancement of salty perception in patients with hyposmia.

## 1. Introduction

The regulation of the body’s homeostasis is strictly related to salt (sodium chloride, NaCl) [1]. An increased salt intake is considered a risk factor for hypertension, stroke, and high blood pressure [2]. Most people usually consume too much salt, in the area of 9–12 g/day [2]. Salt that is normally used for human consumption is refined (table) salt, which is obtained from rock deposits or seawater [3]. In the Mediterranean region, the main source of sodium is related to the sea salt used in many countries as a condiment [3].

The reduction of salt intake plays an important role in improving population health in many countries [2,4]. Two different ways are indicated to reduce the amount of salt intake: replacing the salt with other tastes or increasing the saltiness with the application of salt enhancers such as herbs and spices [4]. Several studies were carried out to identify salt enhancers, compounds which do not have a salty taste per se, but increase the taste intensity using a low amount of salt [5,6]. Other studies showed that the addition of herbs and spices in foods with low salt content enhanced consumer satisfaction, increased food palatability, and at the same time compensated reductions in salt, fat, and energy [7,8]. The use of a specific type of aromatic herbs and spices is often associated with eating habits and cultures of different countries. In fact, in the Mediterranean zone, different herbs and plants such as basil, bay, peppermint, rosemary, and sage are normally used to increase food flavor and taste perception [3,8,9]. Gao and Colleagues suggested the use of Chinese douche, a traditional fermented soya bean product, to improve salty taste and reduce the salt intake [10]. In our previous study [3], we confirmed the important role of Mediterranean aromatic plants (rosemary, myrtle, orange, and saffron) in the enhancement of salt perception and qualified the use of flavored sea salts as a potential strategy to reduce daily salt intake.

Food flavor perception is considered to be a combination of taste, smell, and trigeminal response [11]. The main process of flavor perception is retronasal olfaction [12]. Patients with olfactory deficits usually report less food enjoyment, decreased flavor perceptive capacity, and changes in dietary behavior with weight gain or weight loss [13,14]. In fact, patients with olfactory loss try to compensate for their dysfunctions by using an increased quantity of sweeteners, salt, or spices to obtain a more hedonic gratification through activation of the gustatory and trigeminal pathways [15]. Few data have been reported on the salt intake in patients with hyposmia. Zang and colleagues reported that olfactory function may be associated with a decreased perception in salty taste intensity [16]. In addition, Passàli and Colleagues indicated that patients with olfactory dysfunction use a larger amount of salt, increasing the risk of hypertension [17]. Another study indicated that only one-third of patients with hyposmia used more salt in foods due to a compensatory mechanism in response to olfactory deficits [18]. On the other hand, the mechanism of this increased use of salt is not well-known [18]. A recent study indicated different herbs and spices as a possibility for the improvement of COVID-19-induced anosmia and ageusia [19].

Starting from all of these considerations, the aim of this study was to evaluate for the first time the role of Mediterranean aromatic herbs and spices in the salty taste perception of patients with hyposmia compared to healthy controls. In particular, the salty taste perception of saline solutions from pure salt (S) and different types of commercial flavored sea salt (FS) was assessed in total patients with hyposmia compared to healthy controls. People with hyposmia were divided into patients without (no-COVID) and with a post-acute coronavirus (COVID-19) syndrome (COVID). Leaves and myrtle berries (flavored salt: FS 1), a mixture of Mediterranean herbs and plants (helichrysum, rosemary, liquorice, fennel seeds, and myrtle leaves) (FS 2), oranges and saffron (FS 3) were used as salt flavoring ingredients. Differences in gustatory perception between patients with hyposmia and controls were evaluated considering the rate of the gustatory dimensions of pleasantness, intensity, and familiarity.

## 2. Materials and Methods

### 2.1. Participants

One hundred and forty-eight subjects were enrolled; 35 women and 22 men were patients with hyposmia (mean age ± standard deviation (SD), 35.7 ± 14.1), while 62 women and 29 men were healthy controls (mean age ± SD, 31.4 ± 13.6). Among people with hyposmia, 23 (40%) patients showed a post-acute coronavirus (COVID-19) syndrome. Exclusion criteria were cognitive impairment, head or neck trauma, stroke, chronic/acute rhinosinusitis, neurodegenerative disorders, psychiatric conditions, and any disorder which may interfere with the olfactory and gustatory evaluations, as previously reported [20,21]. None of the participants was taking medications (for allergies or other diseases) for 5 days before the test.

Age (years), weight (kg), and height (m), and body mass index (BMI) were collected for all participants. This study was approved by the “Azienda Ospedaliera Universitaria di Cagliari” Ethical Committee (Protocol number: PG/2018/10157) and was performed according to the Declaration of Helsinki. All subjects gave their written informed consent to participate in the study and received an explanatory statement.

### 2.2. Olfactory Function Assessment

The olfactory function was assessed by means of the Sniffin’ Sticks test (Burghart Messtechnik, Wedel, Germany) which consists of three different tasks: odor threshold (OT), odor discrimination (OD), and odor identification (OI) [20,22,23]. All subjects were only allowed to drink water 1 h before the test and were instructed to avoid any smoking and scented products on the testing day. Sniffin’ Sticks consists of pen-like odor-dispensing devices. All participants were blindfolded for the OT and OD tasks. The primary OT task was evaluated using 16 stepwise dilutions of n-butanol [23]. The OT task was assessed by means of a three-alternative forced-choice task (3AFC) and single-staircase technique [20,22,23]. OT scores varied from 16 (subjects who could perceive the lowest concentration) to 1 (subjects who could not perceive the highest concentration). Secondary, OD tests were assessed using 16 pen-like odor-dispensing devices. In the OD task, three different pens were used: two containing the same odor and the third containing the target one with the 3AFC task. The OD total score is calculated as the sum of correct answers and may range from 0 to 16 [24]. Finally, the OI test was evaluated by 16 common odors with four verbal descriptors and a multiple forced choice format (three distractors and one target) [25]. The total olfactory function (threshold–discrimination–identification: TDI score) was calculated: Values of 16.5 or less indicated anosmia, values between 16.5 and 30.5 indicated hyposmia, and values over 30.5 indicated normosmia [23].

### 2.3. Gustatory Function Assessment

Gustatory function was assessed by means of the “Taste Strips” test (Burghart Messtechnik, Wedel, Germany). The test consists of filter paper strips instilled with four concentrations of each of the following basic taste qualities: sweet, bitter, sour, and salty [26]. Concentrations were: 0.4, 0.2, 0.1, 0.05 g/mL of sucrose for the sweet taste; 0.006, 0.0024, 0.0009, 0.0004 g/mL of quinine hydrochloride for the bitter taste; 0.3, 0.165, 0.09, 0.05 g/mL of citric acid for the sour taste; and 0.25, 0.1, 0.04, 0.016 g/mL of sodium chloride for the salty taste [26]. Drinking water was used as a solvent in each taste modality. Before the test, participants rinsed their mouths with drinking water. The global taste score ranged from 0 to 16 and a score < 9 was considered as hypogeusia [26]. Only subjects with normogeusia were evaluated for the sensory assessment of flavored salts.

### 2.4. Gustatory Stimuli

Pure salt (sodium chloride, purity ≥ 99.5%) was obtained from Sigma-Aldrich (St. Louis, MO, USA). Flavored sea salts FS 1, FS 2, and FS 3 (Table 1) were produced and kindly provided by the “Bresca Dorada s.r.l.” company (Muravera, Italy).

The semi-industrial production of flavored salts was carried out as previously reported [3,27]. Table 1 indicates the composition per 100 g of product of FS, as indicated on the commercial labels. FS were grinded to obtain fine grains and were weighted before the sensory assessment.

### 2.5. Procedures to Assess Taste Pleasantness, Intensity, and Familiarity of Sea Salt Solutions

All participants evaluated the taste dimensions (pleasantness, intensity, and familiarity) of different saline solutions using a self-reported Likert scale [3,24,28]. Saline solutions were obtained by dissolving flavored sea salts in drinking water at the concentrations of 0.1 and 0.04 g/mL, which represented the two intermediate concentrations preliminarily used in the Taste Strips test [26] for the assessment of gustatory function. Control salt solutions were obtained by dissolving an equal weight of salt (NaCl) in drinking water. All saline solutions were prepared and presented at room temperature (23 °C), as previously reported [29]. Aliquots of 2 mL of each saline solution were placed in disposable plastic test tubes and used for sensory assessment. The pleasantness, intensity, and familiarity of saline solutions were evaluated using a 7-point Likert-type scale, which ranged from 0-not at all to 6 (such as 0 = very unpleasant and 6 = very pleasant; 0 = not intense at all and 6 = very intense; 0 = not familiar at all and 6 = very familiar). A value of 3 was considered a neutral point. Before the experiment, participants rinsed their mouths with drinking water. The order of taste stimuli was randomized. During the session, participants evaluated eight different saline solutions.

### 2.6. Statistical Analyses

The normal distribution of data was calculated using the Shapiro-Wilk test. For age, weight, height, BMI, OT, OD, OI, TDI score, sweet, salty, sour, and bitter taste statistically significant differences among the four different groups of subjects (controls, total patients with hyposmia, patients with and without COVID) were performed using One-way ANOVA by the SPSS software version 25 for Windows (IBM, Armonk, NY, USA). While significant differences among the four different groups of subjects (controls, total patients with hyposmia, patients with and without COVID as between factors) in relation to type of salt (pure salt and flavored salts as within factor) were calculated using a two-way repeated measures ANOVA. When two-way repeated measures ANOVAs showed statistically significant interactions, sources of significance were detected by pairwise post-hoc comparisons using the honestly significant difference (HSD) Tukey’s test. In all other cases, pairwise comparisons were calculated by a two-tailed *t*-test with Bonferroni’s correction. Two-way repeated measures ANOVAs were carried out by GraphPad Prism version 9.0.0 for Windows (San Diego, CA, USA). Bivariate correlations were assessed using the Pearson’s correlation coefficient (*r*). Furthermore, an exploratory multivariate linear regression analysis was performed to assess the potential contribution of olfactory function (TDI score) on each taste perception. In the multivariate linear regression analysis, the TDI score was set as a dependent variable, while sweet, salty, sour, and bitter taste were independent variables. All bivariate and multivariate analyses were calculated using SPSS software version 25 for Windows (IBM, Armonk, NY, USA). The significance level was set at *p* < 0.05. Data were expressed as a mean value ± standard deviation (SD).

## 3. Results

### 3.1. Differences in Olfactory and Gustatory Function between Total Patients with Hyposmia, No-COVID, and COVID Patients Compared to Healthy Controls

Table 2 indicated mean values ± SD for age, weight, height, BMI, OT, OD, OI, and TDI score measured in total in patients with hyposmia, COVID, no-COVID, and healthy controls. No significant differences were observed between patients with hyposmia and healthy controls for mean age, weight, height, and BMI (Table 2).

In patients with hyposmia compared to healthy controls, mean values ± SD for OT, OD, OI, and TDI score are shown in Table 2. In total patients with hyposmia, significant decreased scores were observed for OT (*p* < 0.001), OD (*p* < 0.001), OI (*p* < 0.001), and TDI score (*p* < 0.001) compared to healthy controls. Among patients with hyposmia, the no-COVID and COVID ones showed a significant decrease in scores of OT (*p* < 0.001), OD (*p* < 0.001), OI (*p* < 0.001), and TDI score (*p* < 0.001). Interestingly, significant differences were observed for OD and TDI scores between no-COVID versus COVID patients (*p* < 0.01 and *p* < 0.05, respectively).

**Figure 1:**
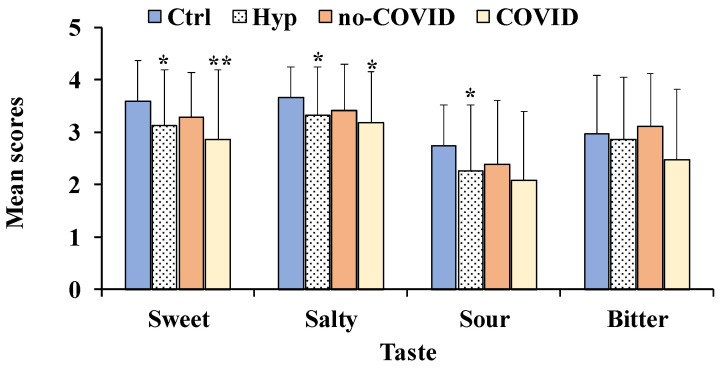
Mean values ± standard deviation of sweet, salty, sour, and bitter taste scores for total patients with hyposmia (Hyp, *n* = 57), subjects without (no-COVID, *n* = 34) or with (COVID, *n* = 23) post-acute Coronavirus Syndrome compared to healthy controls (Ctrl, *n* = 91). For each taste modality (sweet, salty, sour, and bitter) significant differences were: ** = *p* < 0.01 and * = *p* < 0.05 versus Ctrl (One-way ANOVA).

As regards gustatory function (Figure 1), mean values ± SD for sweet, salty, sour, bitter, and total taste scores were 3.2 ± 1.1, 3.3 ± 0.93, 2.3 ± 1.3, 2.9 ± 1.2, 11.6 ± 2.9, respectively in total patients with hyposmia, while in healthy controls the same values were 3.6 ± 0.8, 3.7 ± 0.6, 2.9 ± 1.1, and 12.9 ± 1.7, respectively.

Patients with hyposmia showed a significant impairment in sweet (*p* < 0.05), salty (*p* < 0.05), and sour taste perception (*p* < 0.05) compared to healthy controls (Figure 1). Among total patients with hyposmia, subjects with COVID showed a significant decrease in sweet (*p* < 0.01) and sour taste (*p* < 0.05) perception compared to controls.

### 3.2. Correlations between Olfactory Function and Each Taste Perception

Table 3 showed Pearson’s correlations between TDI score versus each taste modality in all subjects. Low significant correlations emerged between TDI score versus sweet, salty, and sour, while no significant correlations were found between TDI score versus bitter taste (Table 3).

Furthermore, a multivariate linear regression analysis was performed to assess the potential contribution of global olfactory function (TDI score) on each taste modality in all subjects. In the multivariate linear regression analysis, the TDI score was considered as a dependent variable, while sweet, salty, sour, and bitter were independent variables. Significant associations emerged between TDI score versus salty (*p* < 0.01) and sour perception (*p* ≤ 0.001). This model explained 19% of variance (R^2^ = 0.188) for the salty and taste perception (Table 4).

### 3.3. Ratings of Taste Pleasantness, Intensity, and Familiarity for the Saline Solutions Obtained with Normal Sea Salt and Flavored Salts

**Figure 2:**
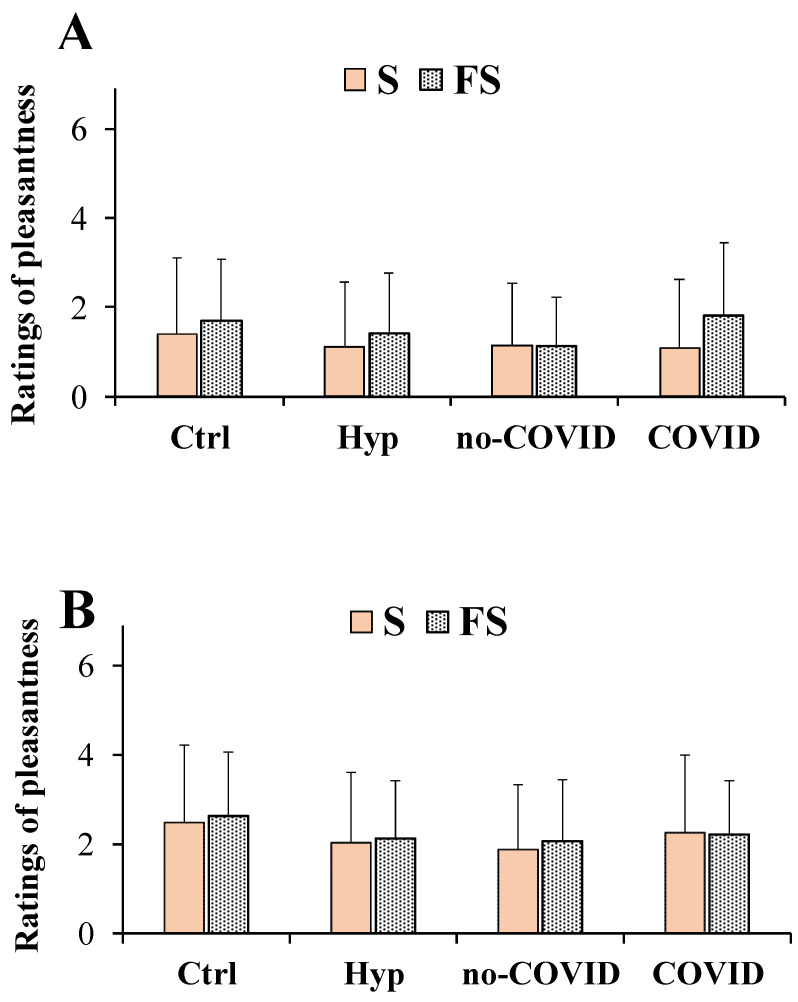
Ratings of pleasantness dimension measured in total patients with hyposmia (Hyp, *n* = 57), subjects without (no-COVID, *n* = 34) or with (COVID, *n* = 23) post-acute coronavirus syndrome compared to healthy controls (Ctrl, *n* = 91), in response to saline solutions obtained with pure sea salt (S) and flavored salts (FS) at the concentrations of 0.1 g/mL (high dose) (**A**) and 0.04 g/mL (low dose) (**B**). Data are presented as mean values and standard deviations. For FS, each value of the taste dimensions was obtained as the mean of participant responses to the three different commercial FS samples. Significant differences among the four different groups of subjects (as between factors) in relation to the type of salt (S and FS as within factor) were calculated using two-way repeated measures ANOVA.

Figure 2 shows the ratings of the pleasantness dimension determined for the saline solutions obtained with S and FS at concentrations of 0.1 g/mL (high dose, Figure 2A) and 0.04 g/mL (low dose, Figure 2B) in total patients with hyposmia, in no-COVID and COVID ones compared to healthy controls. Concerning FS, each value of taste dimensions was obtained as the mean of participant responses to the three different commercial FS samples previously cited.

In general, at both doses, all saline solutions were unpleasant for all subjects, showing low scores (below 3). Moreover, at high doses, normal and flavored salts were perceived as less pleasant than at low doses in all participants. At the high dose for the pleasantness dimension, a two-way ANOVA for repeated measures did not detect any significant salt per groups interaction (F_(3,201)_ = 0.88, *p* > 0.05) (Figure 2A). The main effect of the type of salt (S and FS considered as within-factor) among all groups of subjects (Controls, total patients with hyposmia, no-COVID, and COVID) was statistically significant (F_(1,201)_ = 5.426, *p* < 0.05). In general, FS showed a higher rating of pleasantness dimension than pure salt both in patients with total hyposmia, COVID, and in controls (Figure 2A). Mean values ± SD of pleasantness rating for the FS at the high dose were 1.40 ± 1.37, 1.14 ± 1.09, 1.80 ± 1.65 and 1.71 ± 1.36 in total patients with hyposmia, no-COVID and COVID patients, and controls, respectively (Figure 2A). At the low dose no significant interaction or main effect of factors was observed (F_(3,201)_ = 0.057, *p* > 0.05) for salt per groups and (F_(1,201)_ = 0.630, *p* > 0.05) for type of salt (Figure 2B). Mean values ± SD of pleasantness rating for FS at the concentration of 0.04 g/mL (low dose) were 2.13 ± 1.30, 2.07 ± 1.37, 2.22 ± 1.20, and 2.63 ± 1.44 for patients with hyposmia, no-COVID and COVID patients, and controls, respectively (Figure 2B).

**Figure 3:**
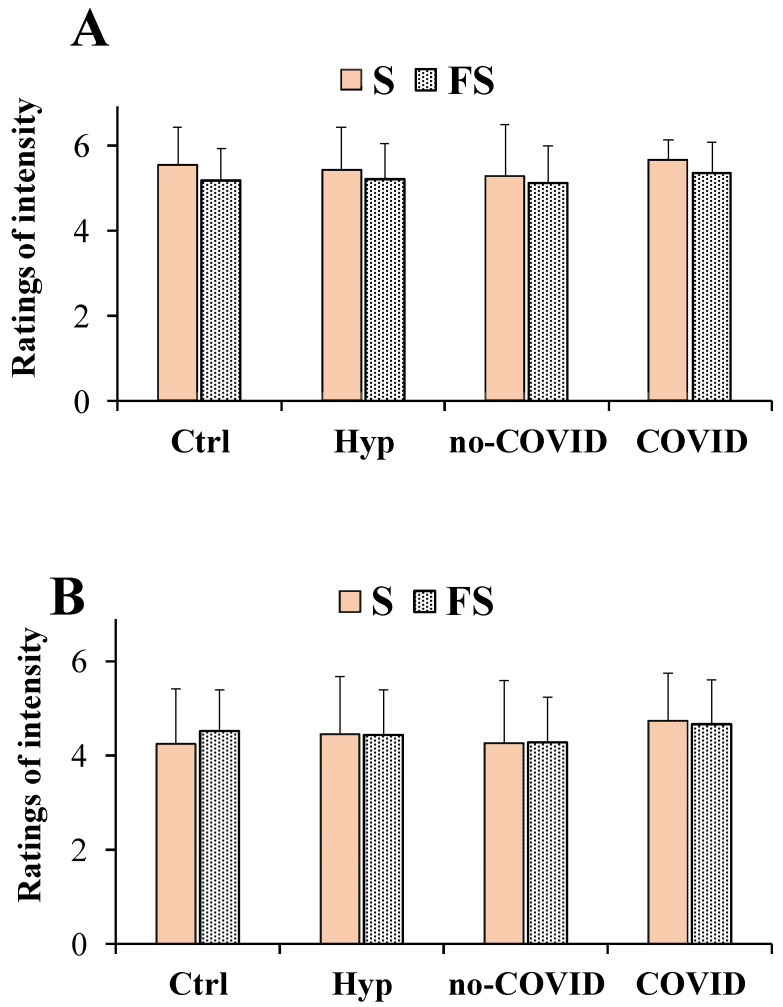
Ratings of intensity dimension in total patients with hyposmia (Hyp, *n* = 57), subjects without (no-COVID, *n* = 34) or with (COVID, *n* = 23) post-acute Coronavirus Syndrome compared to healthy controls (Ctrl, *n* = 91), in response to the saline solutions obtained with pure salt (S) and flavored salts (FS) at the concentrations of 0.1 g/mL (high dose) (**A**) and 0.04 g/mL (low dose) (**B**). Data are presented as mean values and standard deviations. Regarding FS, each value of the taste dimensions was obtained as the mean of participant responses to the three different commercial FS samples. Significant differences among the four different groups of subjects (as between factors) in relation to the type of salt (S and FS as within factor) were calculated using a two-way repeated measures ANOVA.

Figure 3 showed ratings of intensity for the saline solutions obtained with pure salt (S) and flavored sea salt (FS) at concentrations of 0.1 g/mL (high dose, Figure 3A) and 0.04 g/mL (low dose, Figure 3B), considering total patients with hyposmia, no-COVID, and COVID ones.

Regarding the intensity ratings at the high dose, a two-way repeated measures ANOVA did not detect any significant salt per group interaction (F_(3,201)_ = 0.426, *p* > 0.05). The main effect of the type of salt (S and FS considered as with-in-factor) among all groups of subjects (Controls, total patients with hyposmia, no-COVID, and COVID) was statistically significant (F_(1,201)_ = 5.911, *p* < 0.05) (Figure 3A). In general, at high doses, flavored salt was perceived as being slightly less intense than the pure salt both in total patients with hyposmia, COVID, and controls. Mean values ± SD of intensity rating at the high dose in healthy controls were 5.53 ± 0.90 and 5.16 ± 0.75 for S and FS, respectively. At a high dose, S and FS were characterized by a very high score (near to 6) for the intensity dimension. Instead, at a low dose for the intensity ratings, no significant interaction or main effect of factors was observed (F_(3,201)_ = 0.850, *p* > 0.05) for salt per groups and (F_(1,201)_ = 0.372, *p* > 0.05) for type of salt (Figure 3B). However, the intensity was higher for FS than S in healthy controls, while similar scores were interestingly observed in total patients with hyposmia.

**Figure 4:**
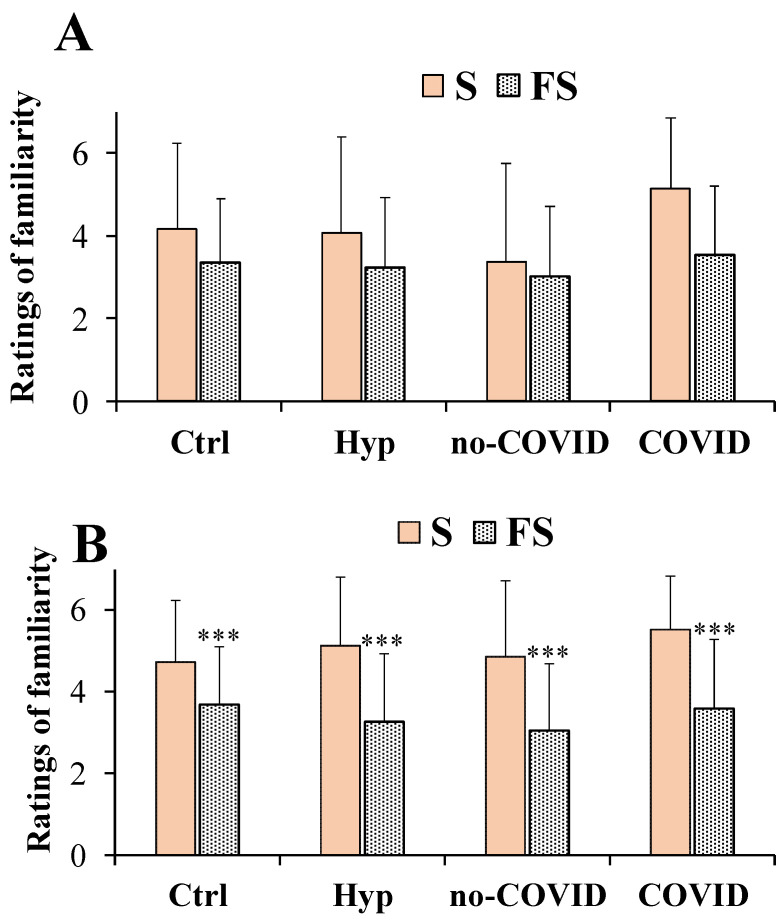
Ratings of familiarity dimension measured in total patients with hyposmia (Hyp, *n* = 57), subjects without (no-COVID, *n* = 34) or with (COVID, *n* = 23) post-acute coronavirus syndrome compared to healthy controls (Ctrl, *n* = 91), in response to saline solutions obtained with pure sea salt (S) and flavored salts (FS) at the concentrations of 0.1 g/mL (high dose) (**A**) and 0.04 g/mL (low dose) (**B**). Data are presented as mean values and standard deviations. For FS, each value of the taste dimensions was obtained as the mean of participant responses to the three different commercial FS samples. Significant differences among the four different groups of subjects (as between factors) in relation to type of salt (S and FS as within factor) were calculated using two-way repeated measures ANOVA. *** = *p* < 0.001 FS versus S.

In general, FS showed a decrease in familiarity ratings at the high (Figure 4A) and low (Figure 4B) doses compared to pure salt in all groups of subjects.

For the familiarity ratings at the high dose, the two-way repeated measures ANOVA did not detect any significant salt per groups interaction (F_(3,201)_ = 1.599, *p* > 0.05) (Figure 4A). The main effect of the type of salt (S and FS considered as with-in-factor) among all groups of subjects (Controls, total patients with hyposmia, no-COVID, and COVID) was statistically significant (F_(1,201)_ = 29.88, *p* < 0.001). Mean values ± SD of S familiarity at the high dose were 4.18 ± 2.07, 4.09 ± 2.29, 3.38 ± 2.39, and 5.13 ± 1.71 for controls, total patients with hyposmia, no-COVID, and COVID patients, respectively. Instead, mean values ± SD of FS at the high dose were 3.35 ± 1.54, 3.22 ± 1.70, 3.54 ± 1.66, and 3.01 ± 1.71 for controls, total patients with hyposmia, no-COVID, and COVID patients, respectively.

At the low dose, for the familiarity ratings, significant interactions and main effects of factors were observed (F_(3,201)_ = 3.194, *p* < 0.05) for salt per groups and (F_(1,201)_ = 123.5, *p* < 0.001) for type of salt (Figure 4B). FS showed lower significant (*p* < 0.001) familiarity ratings compared to S in controls, in total patients with hyposmia, in no-COVID, and in COVID patients.

**Figure 5:**
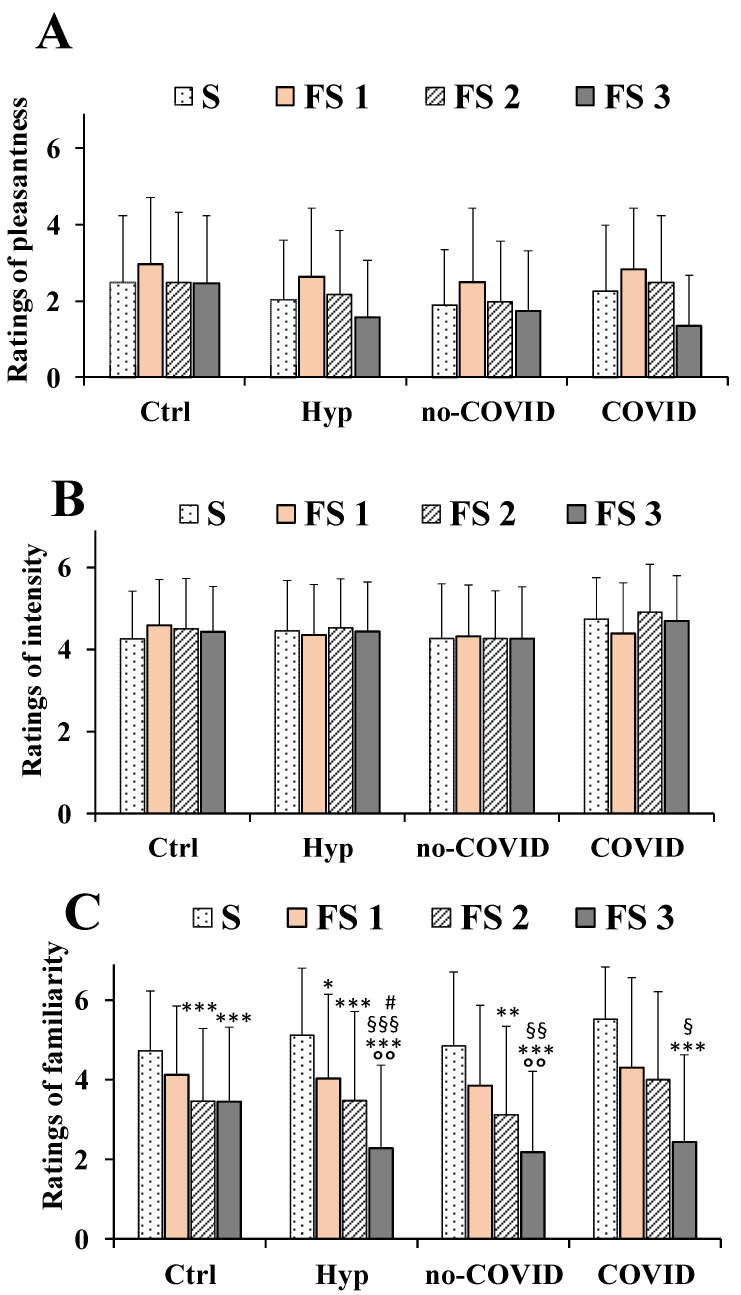
Ratings of pleasantness (**A**), intensity (**B**), and familiarity (**C**) dimensions measured in total patients with hyposmia (Hyp, *n* = 57), subjects without (no-COVID, *n* = 34) or with (COVID, *n* = 23) post-acute Coronavirus Syndrome compared to healthy controls (Ctrl, *n* = 91), in response to saline solutions obtained with pure salt (S) and flavored sea salts of myrtle (FS 1), of Mediterranean herbs/plants (FS 2), and of orange/saffron (FS 3) at the concentrations of 0.04 g/mL. Data are presented as mean values and standard deviations. Significant differences among the four different groups of subjects (as between factors) in relation to type of salt (S, FS 1, FS 2, and FS 3 as within factor) were calculated using a two-way repeated measures ANOVA. °° = *p* < 0.01 versus respective Ctrl; *** = *p* < 0.001, ** = *p* < 0.01, * = *p* < 0.05 versus S; ^§§§^ = *p* < 0.001, ^§§^ = *p* < 0.01, ^§^ = *p* < 0.05 versus FS 1; ^#^ = *p* < 0.05 versus FS 2.

The ratings of pleasantness, intensity, and familiarity dimensions determined for the taste of saline solutions (at the dose of 0.04 g/mL) obtained with the three types of flavored sea salt (FS 1, FS 2, and FS 3) respect to pure salt, are reported for total patients with hyposmia, subjects no-COVID, COVID, and healthy controls in Figure 5.

In general, at the low dose, S, FS 1, FS 2, and FS 3 were similar in taste pleasantness showing scores below 3 (Figure 5A) both in patients with hyposmia and healthy controls. A two-way repeated measures ANOVA did not detect any significant salt per group interaction (F_(9,603)_ = 1.215, *p* > 0.05) for the taste pleasantness. The main effect of the type of salt (S, FS 1, FS 2, and FS 3 considered as within-factor) among all groups of subjects was statistically significant (F_(3,603)_ = 15.39, *p* < 0.001) for the familiarity ratings. Some other differences, unless not significant, were observed among diverse flavored salts, with FS 1 (salt of myrtle) showing the highest scores in the taste pleasantness dimension among all subjects.

In the ratings of familiarity dimension, the two-way repeated measures ANOVA showed significant interaction and the main effect of factors: (F_(9,603)_ = 3.224, *p* < 0.001) for salt per groups and (F_(2.916,586.1)_ = 68.44, *p* < 0.001) for type of salt (Figure 5C). FS 1, FS 2, and FS 3 showed significant lower scores in familiarity dimension than pure salt both in patients with hyposmia and healthy controls (Figure 5C). This difference in familiarity ratings was more marked in patients with hyposmia than in controls, with the following order: FS 3 < FS 2 < FS 1 < S. In particular, FS 3 showed significant lower scores for the familiarity ratings dimension in total patients with hyposmia, No-COVID, and COVID ones (Figure 5C).

With regard to the intensity dimension, the two-way repeated measures ANOVA showed no significant interaction and main effect of factors: (F_(9,603)_ = 0.895, *p* > 0.05) for salt per groups and (F_(2.695,541.7)_ = 0.649, *p* > 0.05) for type of salt (Figure 5B).

In all subjects the taste intensity scores were very similar for all flavored salts compared to pure salt (Figure 5B). In total patients with hyposmia, the intensity ratings were 4.46 ± 1.23, 4.35 ± 1.23, 4.53 ± 1.20, and 4.44 ± 1.21, while values of 4.25 ± 1.17, 4.59 ± 1.12, 4.51 ± 1.22, and 4.43 ± 1.11, were determined for healthy controls for S, FS 1, FS 2, and FS 3, respectively.

## 4. Discussion

Hyposmia, a quantitative olfactory deficit, is considered a reduction in olfactory function [30]. People with hyposmia usually show daily life problems in food intake, dietary behavior, personal hygiene, safety, and sexual behavior [13,14]. Among patients with hyposmia, a decreased salty taste perception has been previously reported [18]. According to the literature, in our study patients with hyposmia showed a significant decrease in sweet, salty, and sour taste perception compared to healthy controls, while no significant differences for bitter perception were found. In previous studies, patients with hyposmia showed an increase in salt consumption [18,31,32]. Similarly, we demonstrated that patients with hyposmia exhibited alterations of sweet, salty, and sour taste preference as a consequence of the damage in flavor perception. Our data indicated significant positive correlations between global olfactory function (TDI score) versus salty and sour perception. These correlations may be due to the interaction between gustatory, olfactory, and trigeminal function, as reported in previous studies [16,21]. An association between olfactory function and food perception was previously observed by Zang and colleagues, suggesting that changes in odor perception may induce differences in food intake [16].

In general, patients with hyposmia in order to obtain a gustatory gratification tray to compensate the decrease of flavor perception using salts and spices [8,9]. Previous studies suggested that the addition of herbs and spices could be used as an alternative of or in combination with the salt in food, as a simple strategy in the reduction of the salt amount in the diet [8,9]. In a previous study, we demonstrated the important role of Mediterranean herbs and spices in the enhancement of the salty perception in healthy controls and qualified the use of flavored sea salts as a potential strategy to reduce the daily salt intake [3]. The present study is focused on the role of aromatic herbs and spices in the enhancement of salty perception in patients with hyposmia, in order to confirm their potential use for the improvement of the flavor perceptive capacity in people with olfactory deficits. The salty taste dimensions (pleasantness, intensity, and familiarity) of saline solutions obtained with pure salt and several flavored sea salts were assessed in patients with hyposmia in order to evaluate the role of flavoring in salty taste perception. Commercial sea salts used in this study were flavored with a mixture of herbs, spices, and fruits used in the diet and in the popular Mediterranean tradition such as myrtle, fennel, rosemary, helichrysum, liquorice, saffron, and orange [3,27,33,34,35].

Our results showed that flavored sea salts and pure salt were similar in taste pleasantness both in patients with hyposmia and healthy controls (considering the mean value of participant’ responses for the three different commercial samples). At both doses no significant differences in pleasantness scores between patients with hyposmia and healthy controls were observed. At a dose of 0.1 g/mL, it was difficult to appreciate chemosensory differences in the taste dimensions of the saline solutions due to the high concentrations used, thus our attention was focused on low doses (0.04 g/mL). In the literature it is known that salt at medium-low concentrations is usually perceived as pleasant and palatable [1], while at high doses it activates aversive taste pathways [36]. In fact, in our study, for patients with hyposmia at the dose of 0.04 g/mL it was possible to evaluate more accurate differences in salty perception. Interestingly, in patients with hyposmia, flavored sea salt exhibited an equal intensity to that of the pure sea salt. This finding is very fascinating since flavored salt contains approximately 15% less sodium chloride, confirming the role of spices and herbs in the enhancement of the salty perception in patients with hyposmia. Moreover, flavored salt was less familiar than pure sea salt in total patients with hyposmia, showing significant lower familiarity ratings compared to controls. Among flavored salts, in patients with hyposmia, FS 3 (the salt of oranges and saffron) emerged as the most interesting in potentiating saltiness perception (considering the 30% reduction of salt weight). However, FS 3 showed the lowest scores in taste pleasantness and familiarity (indicating a less common taste). These results indicated that the saline solutions obtained with flavored salts, containing approximately 6% to 30% of flavoring extract, were perceived as equally intense as pure salt in total patients with hyposmia and patients with or without COVID.

There is actually great interest in the dietary use of fresh herbs and spices due to their ability to impart distinctive aromas, which may modulate the salty taste perception [9]. Herbs and spices provide proteins, fiber, volatile components (essential oils), vitamins, minerals, phytochemicals, and contribute significantly to the promotion of human health due to their different beneficial properties (antioxidant activity, anti-carcinogenic activity, and the prevention of cardiovascular and neurodegenerative diseases) [9,37]. Different previous studies confirmed the use of herbs and spices as an excellent strategy for the reduction of the salt amount in food products [8,9,37,38]. However, the use of aromatic herbs and spices is often associated to the culinary and cultural habits of subjects [3,10]. Moreover, it is important to note that the use of aromatic herbs and spices rich in phytochemicals with anti-inflammatory effects has also been demonstrated as a potential strategy for the treatment of hyposmia and ageusia [19].

Flavor is the combination of soluble and non-volatile compounds, and of volatile compounds perceived through the retronasal smell, and chemical sensations through the trigeminal nerve [39,40,41]. Polar components (phenols, sugars, and amino acids) previously identified in flavored salts [27] may play an important role in the modulation of the salty perception. In particular, polar compounds of plant extracts incorporated in the flavored sea salt are soluble in water and may modulate the taste perception, while volatile compounds previously identified in the tested flavored sea salts [3] could be responsible for aromatic properties through the retronasal olfactory function. The flavor perception is a complex process, and mechanisms involved in the interaction between flavored salts and receptors in the oronasal cavity are not yet well known. The strengths of this study are the combination of olfactory and gustatory objective tests such as Sniffin’ Sticks and Taste Strips with a hedonic assessment of taste stimuli such as pleasantness, intensity, and familiarity using a Likert scale. Usually, pleasantness, intensity, and familiarity ratings of a food may be regulated by different parameters such as the subject’s experience and expectations, dietary behavior, cultural tradition, and homeostatic balance [42]. In our study, patients with hyposmia showed a decrease in orthonasal olfactory function, but their retronasal olfactory function and trigeminal perception was probably still maintained. In the literature, it is well known that food perception is considered a sequential process which starts in the oronasal cavity with peripheral sensory mechanisms, then involves brain areas with cross-modal interactions and multisensory integrations [41,42]. The enhancement of saltiness perception observed in total patients with hyposmia with the salt of oranges and saffron (FS 3) is probably correlated to the specific complex composition, being rich in limonene, sugars and amino acids from orange flavedo [3], water-soluble carotenoids (crocetin and crocins), and fat-soluble carotenoids (carotene, lycopene, and zeaxanthin) [43].

In addition, among patients with hyposmia, we also considered differences between no-COVID and COVID subjects. In the literature it has been reported that 41–62% of COVID patients showed olfactory and gustatory deficits [44,45,46]. A possible explanation of this data is that it may be due to a consequence of the damage in flavor perception. Gustatory deficits in COVID patients could be considered to be a consequence of upper respiratory tract infection, damage of the taste papillae, and post viral cranial nerve damage [47]. Moreover, our data demonstrated that COVID patients showed higher scores, unless not significant, of intensity ratings compared to no-COVID ones in response to both flavored salt and natural sea salt. This alteration in salty taste in COVID patients may be due to changes in saliva composition and in sodium homeostasis as reported by Asadi et al. [44].

This study has some limitations. We did not objectively evaluate the effect of food matrices and temperature in saltiness perception, as previously reported for the assessment of preferences in different concentrations of monosodium glutamate [48].

## 5. Conclusions

Saline solutions obtained from flavored salts were less familiar and equally intense, with similar scores of the pleasantness dimension, than pure sea salt in patients with hyposmia. Our data confirmed that the addition of Mediterranean herbs and spices to sea salt enhanced the salty taste perception in patients with olfactory dysfunction, highlighting the potential role of flavored salts for the reduction of salt intake in their daily diet and for a better gustatory perception of the food. Further studies are necessary to determine the best ratio and type of mixture of spices and aromatic herbs in the flavored salt for clinical application in the daily diet of patients with hyposmia, together with the evaluation of the specific substances that could affect the salty sensation.

## Figures and Tables

**Table 1:** Composition and digital images of different commercial flavored sea salts indicated per 100 g [3].

Sample		Commercial Name	Composition as Indicated on the Labels (100 g of Product)
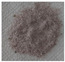	FS 1	Salt of myrtle	Sardinian sea salt, myrtle essential oil, extract of myrtle (water, berries and leaves of *Myrtus communis*) (6%)
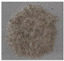	FS 2	Salt of Mediterranean herbs and plants	Sardinian sea salt, extract of mixed herbs (helichrysum, rosemary, liquorice, fennel seeds, and myrtle leaves) (15%)
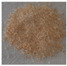	FS 3	Salt of oranges and saffron	Sardinian sea salt, extract of oranges and saffron (peels and juice of oranges fruits, *Crocus sativus*) (30%)

**Table 2:** Demographic and clinical features of total patients with hyposmia, patients with (COVID) or without (no-COVID) post-acute coronavirus syndrome, compared to healthy controls.

Parameters	Control (*n* = 91)	Hyposmia (*n* = 57)	No-COVID (*n* = 34)	COVID (*n* = 23)
Mean age	31.4 ± 13.6	35.7 ± 14.8	36.3 ± 16.1	34.9 ± 13.1
Sex	62 W/29 M	35 W/22 M	21 W/13 M	14 W/9 M
Weight (kg)	62.9 ± 12.7	65.8 ± 16.1	63.9 ± 17.7	68.6 ± 17.9
Height (m)	1.6 ± 0.1	1.6 ± 0.2	1.6 ± 0.1	1.6 ± 0.1
BMI	23.2 ± 3.8	23.9 ± 4.8	23.3 ± 3.8	24.7 ± 5.8
OT	10.2 ± 4.2	3.7 ± 2.3 ***	3.6 ± 1.9 ***	3.6 ± 2.6 ***
OD	12.4 ± 1.3	10.1 ± 2.5 ***	10.8 ± 1.8 ***	9.1 ± 3.1 *** ^§§^
OI	13.2 ± 1.3	11.6 ± 2.1 ***	11.9 ± 1.4 ***	11.1 ± 2.7 ***
TDI score	35.8 ± 3.9	25.5 ± 4.3 ***	26.4 ± 2.6 ***	23.8 ± 5.7 *** ^§^

Legend: W: women; M: men; BMI = body mass index; OT = odor threshold; OD = odor discrimination; OI = odor identification; TDI score = threshold, discrimination, and identification score. Significant differences between groups (controls, patients with hyposmia, no-COVID, and COVID) was assessed by one–way analysis of variance (One-way ANOVA). *** = *p* < 0.001 compared to controls; ^§^ = *p* < 0.05 no-COVID versus COVID; ^§§^ = *p* < 0.01 no-COVID versus COVID.

**Table 3:** Pearson’s correlations between TDI score versus gustatory function in all subjects.

Gustatory Function	TDI Score
Sweet	*r* = 0.243, *p* < 0.01
Salty	*r* = 0.285, *p* < 0.01
Sour	*r* = 0.321, *p* < 0.01
Bitter	*r* = 0.001, *p* > 0.05

Legend: TDI score = threshold, discrimination, and identification score; *r* = Pearson’s coefficient.

**Table 4:** Multivariate linear regression analyses in all subjects. TDI score as dependent variable.

Parameters	Unstandardized Coefficients		Standard Coefficients		
	B	Std Error	β	*t*	Significance
Sweet	0.917	0.607	0.132	1.512	*p* > 0.05
Salty	2.056	0.707	0.242	2.910	*p* < 0.01
Sour	1.733	0.525	0.274	3.300	*p* < 0.01
Bitter	−0.613	0.467	−0.109	−1.312	*p* > 0.05

Legend: B = unstandardized coefficient for each predictor variable; β = standardized coefficient which gives a measure of the variable contribution; *t* = *t*-values which indicate whether the predictor’s regression coefficient is significant.

## Data Availability

Not applicable.
